# Emerging Insights into Brevetoxicosis in Sea Turtles

**DOI:** 10.3390/ani14070991

**Published:** 2024-03-22

**Authors:** Remco A. Nederlof, Dion van der Veen, Justin R. Perrault, Robin Bast, Heather W. Barron, Jaco Bakker

**Affiliations:** 1 Independent Researcher, 2861 XZ Bergambacht, The Netherlands; 2 Independent Researcher, 1406 LP Bussum, The Netherlands; dionvanderveen3344@gmail.com; 3 Loggerhead Marinelife Center, Juno Beach, FL 33408, USA; jperrault@marinelife.org (J.R.P.); hbarron@marinelife.org (H.W.B.); 4 Clinic for the Rehabilitation of Wildlife, Inc., Sanibel, FL 33957, USA; rbast@crowclinic.org; 5 Animal Science Department, Biomedical Primate Research Centre, Lange Kleiweg 161, 2288 GJ Rijswijk, The Netherlands; bakker@bprc.nl

**Keywords:** *Karenia brevis*, brevetoxin, red tide, harmful algal blooms, climate change, mortality, biotoxin, exposure, treatment, intravenous lipid emulsion

## Abstract

**Simple Summary:**

Florida red tides are large algal blooms of the toxic organism *Karenia brevis* and can have severe ecological impacts along the Gulf Coast regions of Florida, USA. *K. brevis* blooms produce potent neurotoxins, known as brevetoxins, which are known to cause disease and mortality in various marine species, including sea turtles. This review examines the impact of red tides on sea turtle health by discussing *K. brevis* blooms in general, as well as the toxin exposure routes and mechanisms of disease. Diagnostic and treatment options are also discussed. Significant research efforts have focused on developing improved therapeutic interventions, and intravenous lipid emulsion therapy has proven highly effective at rapidly alleviating symptoms and accelerating brevetoxin removal from sea turtles. This review synthesizes the current scientific understanding of red tide impacts on threatened and endangered sea turtle health. Continued investigations of outstanding knowledge gaps may help mitigate the threat of harmful algal blooms on sea turtles in the future and may contribute to the conservation of sea turtles.

**Abstract:**

This review summarizes the current understanding of how brevetoxins, produced by *Karenia brevis* during harmful algal blooms, impact sea turtle health. Sea turtles may be exposed to brevetoxins through ingestion, inhalation, maternal transfer, and potentially absorption through the skin. Brevetoxins bind to voltage-gated sodium channels in the central nervous system, disrupting cellular function and inducing neurological symptoms in affected sea turtles. Moreover, the current evidence suggests a broader and longer-term impact on sea turtle health beyond what is seen during stranding events. Diagnosis relies on the detection of brevetoxins in tissues and plasma from stranded turtles. The current treatment of choice, intravenous lipid emulsion therapy, may rapidly reduce symptoms and brevetoxin concentrations, improving survival rates. Monitoring, prevention, and control strategies for harmful algal blooms are discussed. However, as the frequency and severity of blooms are expected to increase due to climate change and increased environmental pollution, continued research is needed to better understand the sublethal effects of brevetoxins on sea turtles and the impact on hatchlings, as well as the pharmacokinetic mechanisms underlying brevetoxicosis. Moreover, research into the optimization of treatments may help to protect endangered sea turtle populations in the face of this growing threat.

## 1. Introduction

Generally, the term ‘red tide’ refers to the discoloration of seawater due to a harmful algal bloom (HAB), usually caused by dinoflagellates. In the Gulf of Mexico, red tide refers to an HAB of the toxic dinoflagellate *Karenia brevis*, which may appear as reddish discoloration of the water [1]. These blooms are most frequently reported on the Florida West Coast, but blooms also occur in Texas and can even reach as far north as North Carolina’s Atlantic Coast [1,2,3]. Increases in the frequency, duration, range, and impact of HABs have been reported [4,5,6] and have been linked to climate change in conjunction with anthropogenic pressures [7,8]. However, these trends are poorly understood, and reported increases may be attributable to intensified monitoring and bloom impacts [9]. Therefore, HAB patterns should be considered regionally and at the species level. 

The causative organism of red tide HABs, *K. brevis*, previously known as *Gymnodinium breve* and *Ptychodiscus brevis*, is an unarmored (i.e., without thecal plates), small-to-medium sized (18–45 μm wide), photosynthetic marine dinoflagellate that produces neurotoxins referred to as brevetoxins [10,11,12]. Brevetoxins are cyclic, lipophilic, poly-ether compounds that are differentiated into two distinct backbone structures: type A with 10 rings and type B with 11 rings. There are as many as 14 polyether brevetoxin (PbTx) compounds, of which the compounds PbTx-1, PbTx-2, and PbTx-3 are most abundantly present during *K. brevis* blooms [13]. The parent algal toxins, PbTx-1 and PbTx-2, undergo metabolic changes that produce a suite of PbTx congeners as the bloom matures [13,14]. PbTx-2 is the predominant intracellular compound, whereas its primary reduction product, PbTx-3, is formed after cellular lysis and is subsequently released into the water column. It has been demonstrated that these extracellular toxins may persist in the water column even after *K. brevis* cell counts have fallen below detectable concentrations [13].

Natural background concentrations of 1000 *K. brevis* cells per liter of seawater result in potential brevetoxin concentrations ranging from 0 to 30 ng/L PbTx without any reported adverse health effects [12]. Concentrations of ~100 ng/L PbTx have been demonstrated to cause filter-feeding shellfish to become toxic for human consumption. The acute toxicity threshold for aquatic animals likely varies for the exposed animal species and may further depend on the route of exposure and the proportions of the various congeners involved [13].

Several mass mortality events of fishes, sea birds, marine mammals, and sea turtles have been attributed to brevetoxin exposure [15,16,17,18,19,20]. While sea turtles are generally considered to be resilient in the face of physical trauma, they are susceptible to chemical insults, and *K. brevis* HABs are estimated to account for 7.1% of loggerhead (*Caretta caretta*), 1.6% of green (*Chelonia mydas*), and 17.7% of Kemp’s ridley (*Lepidochelys kempii*) sea turtle strandings along the Florida West Coast [15]. Moreover, stranded sea turtles reflect a small proportion of all affected animals, as it is likely that many sea turtles die at sea and never strand [21,22]. 

The impact of brevetoxins on these vulnerable animals may extend beyond direct toxicity, as biotoxins have been suggested to act as tumor promotors [23]. Nutrient-rich areas, which predispose local ecosystems to HAB formation, have been correlated with the prevalence of fibropapillomatosis, a tumor-forming disease linked to a herpesvirus that is predominantly found in areas of poor environmental quality [24]. Moreover, a positive correlation was observed between the total number of fibropapillomatosis lesions and plasma brevetoxin concentrations [25]. Although these observations suggest that brevetoxins may play a role in the development of fibropapillomatosis in sea turtles, this relationship is unlikely to be directly causative, as the geographic distribution of fibropapillomatosis far exceeds the area in which red tides occur. Further research is warranted to clarify whether there truly is a relationship between brevetoxin exposure and the development of fibropapillomatosis in sea turtles and, if there is, to elucidate the underlying pathological mechanisms.

## 2. Harmful Algal Blooms

### 2.1. Impact of HABs

A variety of HAB species are found in Florida’s fresh- and saltwater systems and environments, with many species producing toxins that may impact local ecosystems during HAB events [26]. As a result, sea turtles may be exposed to a variety of different toxins simultaneously, even in the absence of bloom events [27,28,29,30]. Of the large variety of toxins produced, brevetoxins have been considered to be one of the most common chemical stressors impacting the coastal and marine ecosystems of South Florida [13].

In humans, brevetoxins are causative of neurotoxic shellfish poisoning (NSP) and are associated with gastrointestinal and neurologic signs following ingestion or a reversible upper respiratory syndrome following inhalation of toxin-containing aerosols [31,32]. Aerosolized brevetoxin respiratory irritation in humans may involve a nonproductive cough, conjunctivitis, rhinorrhea, and sneezing, with wheezing also reported in asthmatic patients [33]. These symptoms may persist for three to four days [34]. The onset of NSP symptoms is rapid and may include nausea, abdominal pain, diarrhea, paresthesia, vertigo, ataxia, and hot–cold temperature reversal. In extreme cases, there may be convulsions and a need for respiratory support [33]. Gastrointestinal emergency room visits have been estimated to increase by as much as 40% during active bloom periods, further indicating the widespread impact of *K. brevis* blooms on human health [35]. 

Whole marine ecosystems may suffer long-lasting effects from *K. brevis* blooms, as mass mortality events add to the environmental stress when the decomposing biomass depletes oxygen from the water. Benthic organisms, such as sea grass and patch reef communities, may require years to recover from such events [36,37]. 

It is important to note that animals are not only exposed to toxins during active blooms but animals may also be exposed to sublethal concentrations of brevetoxins, as well as a variety of other algal toxins, such as saxitoxins and okadaic acid, during non-bloom periods [38]. Not only can toxins produced by marine HABs co-occur, but toxins produced by freshwater HABs may also contaminate the marine environment. This was observed in 2018, when, during a *K. brevis* HAB, microcystins produced by freshwater *Microcystis* spp. flowed from the Caloosahatchee River into the marine environment, leading to detectable concentrations of both brevetoxins and microcystins in the seawater [39].

### 2.2. Occurrence and Distribution of Karenia Blooms

Although background concentrations of *K. brevis* have been determined to be at around 1000 cells per liter of seawater [12], *K. brevis* HABs are frequently defined as cell densities of ≥100,000 cells/L, as fish kills and respiratory issues in humans are observed at this concentration [12,40]. During severe blooms, cell counts may reach as high as 100 million cells/L [40].

These *K. brevis* blooms are a recurrent problem in the United States along the Gulf of Mexico. The west coast of Florida is near-annually affected by these blooms, with some blooms lasting over one year [12]. From 2000 to 2020, red tide blooms were present for 7 ± 3 months per year on average, although blooms lasted anywhere from 1 to 17 months [41].

*K. brevis* blooms can form between 18 and 74 km offshore on the mid-shelf [12]. This formation depends on a variety of factors, among which nutrient availability, water temperature, and salinity play a key role [12]. It has been determined that *K. brevis* does not grow well at low salinities, and as such, blooms may not spread to the upper reaches of Florida’s West Coast estuaries [12]. *K. brevis* grows slowly compared with many other phytoplankton species, with cell counts doubling every three to five days. As a result, explosive growth does not typically occur. Rather, rapid increases in cell count in the water column are likely due to the transport of cells from other areas by tides or currents [12,42]. Moreover, currents, such as the Florida Current and the Gulf Stream, may carry blooms to Florida’s Atlantic Coast and as far north as North Carolina’s Atlantic Coast, increasing the range of impact of these blooms [1,2].

The factors involved in the dissipation of *K. brevis* blooms are not well understood. In many HABs, low nutrient levels are an important reason for the dissipation of a bloom. However, this may be less important for *K. brevis*, as it is adapted to low-nutrient oligotrophic waters [12].

It is thought that human activity generates excessive nutrient runoff, which may result in bloom conditions. It has been determined that a variety of organic compounds, chelators, trace minerals, vitamins, and sulfides play a vital role in the growth of *K. brevis* [12] and that eutrophication increases the probability of HABs forming [36]. An abundance of these compounds, as well as the occurrence of eutrophication, has been associated with increased levels of industry, urbanization, agriculture, fishing activity, and increased shipping activity [36,37,43].

Alongside direct anthropogenic impact, climate change may also influence HABs in terms of geographical distribution, timing, and frequency of occurrence [44]. The death of 12 rough-toothed dolphins (*Steno bredanensis*) in the Canary Islands in 2008 was attributed to brevetoxicosis, signaling the first mass mortality event in European waters. Although the exact source of the brevetoxins could not be determined, *K. brevis* has been recorded in central eastern Atlantic Ocean waters since 2013, likely conditioned by global climate change [45,46]. As a result, changes in the frequency and intensity of HABs due to anthropogenic and climatic changes are expected to impact benthic ecosystems, ultimately affecting all aspects of One Health on a worldwide scale [36,47].

### 2.3. HAB Monitoring, Prevention, and Control Strategies

#### 2.3.1. Monitoring

The integration of data obtained from remote sensing satellite systems, molecular probes, automated toxin or HAB species monitoring buoys, and meteorological data contributes to the early detection and monitoring of HABs [48,49]. Furthermore, currents, deep ocean eddies, and upwelling winds have been identified as conditions that facilitate the delivery of *K. brevis* blooms to nearshore waters [43,50]. By combining local expertise in hydrobiology and hydrography with the aforementioned data, the displacement of HABs can be predicted, and the conditions favorable for HAB development may be identified and monitored [49,50]. Monitoring technology for bloom initiation has made considerable progress with the identification of conditions responsible for bloom initiation [51]. As such, monitoring efforts should focus on known physical conditions that favor or suppress HAB initiation in order to respond to HABs in a timely fashion.

#### 2.3.2. Prevention

Preventive measures aim to keep HABs from occurring or directly impacting a particular resource. Although large advances have been made, much is still unknown about HAB formation, making it difficult to regulate or control factors influencing their formation. Some factors that influence HAB occurrence, such as meteorologic conditions and water salinity, may not be feasibly controlled [52,53]. Additionally, practical considerations may hinder the application of HAB control measures, as HABs may cover thousands of square kilometers and extend as far as 50 m down into the water column [12].

The proliferation of *K. brevis* cells has occurred following increased nitrogenous input from eutrophicated freshwater lakes, demonstrating that anthropogenic pollution plays a role in the observed increases in HAB occurrence [54,55]. As a result, controlling the freshwater flow and total nutrient input into coastal waters may reduce the occurrence of HABs [56]. Such measures may include sewage reduction strategies, reduced fertilizer usage, or the prevention of nitrogen and phosphorus reaching bodies of water [57,58]. Non-polluted freshwater input dilutes pollutants and reduces the stratification of coastal waters, which may be beneficial, as the occurrence of stratification has been associated with dinoflagellate growth. Moreover, restrictions on activities that may result in the transfer of HAB species into environments where they do not naturally occur may aim to prevent the spread of HAB species [52]. The role of the germination of dinoflagellate cysts in the initiation of bloom events and control measures aimed at this life stage has been understudied. As such, future research may focus on a control approach that incorporates the management of the cystic life stage of *K. brevis* [48,59].

#### 2.3.3. Control Strategies

In order to appropriately control HABs, the financial and ecological implications of applied control measures need to be weighed against the impact of the bloom. Other than the impacts of the control strategy, the ecological consequences of controlling these dinoflagellates should be considered carefully. Microalgae, such as *K. brevis*, serve as primary producers within the coastal ecosystem and form a nutrient source for organisms in higher trophic levels. However, at the scale of HABs, microalgae may disrupt the ecosystem’s balance and negatively affect all trophic levels within the ecosystem [60]. Selected control methods should be appropriate for the stage and scale of the bloom, as few methods may be applied on a large scale due to ecological impacts or practical restraints. A combination of measures may need to be considered to combat large-scale oceanic HABs.

Few HAB control studies have focused on *K. brevis*, but some nanoparticles [61], chemicals [62], algicidal bacteria [63,64], protistan grazers [65], algae [66], and biological algicides have been evaluated [67]. 

A multitude of physical control methods has been investigated, but physical methods are generally costly and slow, making them unsuitable for large blooms. Methods may include the large-scale manipulation of water composition aiming to reduce stratification [52], centrifugal or magnetic treatment of water [68], ultraviolet radiation [68], or application of an air extraction method to induce the attachment of microbubbles to algae, causing them to float and be readily collected at the water surface [69].

A relatively safe strategy involves the usage of clay particles that flocculate with algal cells, forming aggregates that may trap or lyse cells [48]. Additionally, clay particles may reduce the amount of toxins in the water column through adsorption [70]. Clay flocculation can be applied to large blooms and is the only established, effective measure to control HABs in the ocean [48,71]. Although non-toxic and inexpensive, research should focus on identifying clay modifiers that are effective on *K. brevis* while reducing the impact of flocculation on benthic marine ecosystems. Phosphatic clays, which have been demonstrated to be effective against *K. brevis*, have been evaluated on a moderate scale, but field applications have not been undertaken due to environmental concerns [56,72]. Moreover, a reduction in planktonic community components has been demonstrated with the usage of recommended clay concentrations. Future research may focus on the long-term impact of frequent clay flocculation on marine ecosystems [56].

Biosurfactants, such as those produced by *Pseudomonas aeruginosa*, have been demonstrated to kill *K. brevis* cells at low concentrations of 5 μg/L. Further research may focus on the environmental impacts of this strategy, but these concentrations were considered to be safe for *Daphnia*, a common species used for toxicity tests [73]. An additional benefit is the high biodegradability of biosurfactants, reducing environmental risks [74]. Moreover, biosurfactants show promising algicidal effects and biodegradation efficiency when used synergistically with clay particles, allowing for an improved cell removal rate and a reduction in the amount of clay required [48,75,76,77].

Other biochemical strategies may involve the use of engineered nanoparticles that may cause cell death through a multitude of mechanisms [78]. These particles have been used in vitro to inhibit *K. brevis*, although risk assessment studies are currently lacking [61].

Algicides have been used in the past to control *K. brevis* blooms. Copper sulfate has been demonstrated to be effective, but this method was deemed expensive, and the effect lasted briefly. More importantly, the risk of broad toxicity to marine ecosystems using this strategy is intolerable [79]. Ozone has been observed to effectively destroy *K. brevis* cells and was able to reduce free brevetoxins in seawater, but more research is required to determine whether this strategy is viable on a large scale [62].

Biological HAB control methods have the potential to be highly effective, but the associated overarching biosafety risks and ethical concerns make them unattractive as HAB control strategies [48]. All biological measures should aim to be specific to *K. brevis* and thoroughly evaluated for ecological impacts. Organisms that feed on dinoflagellates may play a vital role in the termination of naturally occurring HABs [80]. *K. brevis*-specific grazers may be identified and investigated for their usage in mitigating the impact of *K. brevis* HABs. 

Algicidal bacteria may directly lyse HAB cells, cause cell death through excreted compounds, or may outcompete HAB species [81]. Most algicidal bacteria are algae species-specific, and research may focus on identifying bacteria that inhibit *K. brevis* growth [48].

Algicides produced by micro- and macroalgae may inhibit algal bloom growth, including dinoflagellates, while being non-toxic to other organisms [48,82]. Biochemical compounds produced by plants and microorganisms can influence the reproduction of HAB species and constitute a potential low-cost and environmentally friendly method of HAB reduction [83]. One of these biological algicides, aponin, has been evaluated in *K. brevis* and inhibited growth at concentrations of 3 mL/L [67].

Actinomycetes, parasitic organisms, and viruses show potential for *K. brevis* control, but information on target specificity and ecological impacts is lacking [48,84,85,86,87,88,89].

The genetic modification of *K. brevis* may allow for the creation of strains with altered environmental tolerances, reproductive characteristics, or impaired toxin production [52]. Similar to other strategies, concerns about unforeseen consequences of large-scale applications persist.

## 3. Brevetoxin Exposure

### 3.1. Ingestion

The primary route of exposure of sea turtles to brevetoxins is likely by means of ingestion through drinking seawater or through foraging on contaminated prey. Brevetoxins, being lipophilic compounds, have been demonstrated to pass through the buccal mucosa, gut, and blood–brain barrier [90]. Exposure through food items may be particularly high as a result of bioaccumulation and biomagnification. Seagrasses, crustaceans, and fish accumulate high concentrations of toxins in their tissues, which can persist after a *K. brevis* bloom has dissipated [17,91,92].

Fish accumulate brevetoxins by the ingestion of viable *K. brevis* cells and contaminated prey, as well as the direct absorption of brevetoxins across gill membranes [93]. The ingestion of contaminated fish was implicated in a mass mortality event of bottlenose dolphins (*Tursiops truncatus*) in 2004 [17]. Although the diet of both loggerheads and Kemp’s ridleys primarily consists of crustaceans and mollusks, the occasional ingestion of fish may constitute an additional source of brevetoxin exposure for these species [94].

Filter-feeding invertebrates may also accumulate toxins, as is well known from NSP in humans. Oysters rapidly accumulate PbTx metabolites, whereas clams accumulate toxins at a slower rate with longer persistence. This difference likely reflects the higher filtration rate of oysters. Shellfish have been reported to remain toxic for up to four weeks following an HAB and may act as a continued source of exposure [13]. Tunicates have also been observed to accumulate brevetoxins [95]. Secondary consumers, such as fish, crabs, and gastropods, may accumulate brevetoxins by feeding on suspension- and deposit-feeding benthic organisms [96]. Juvenile loggerheads are primarily carnivorous [97,98] and feed on a variety of sessile or slow-moving small animals, such as hydrozoans, jellyfish, bryozoans, gastropods, polychaetes, copepods, and, to a lesser extent, on fishes, crabs, shrimp, seagrasses, algae, and filamentous cyanobacteria [98]. Adult and subadult loggerheads primarily feed on crabs, fishes, gastropods, bivalves, and seagrass [99]. Juvenile Kemp’s ridleys are primarily surface feeders, feeding on gastropods, algae, and, to a lesser extent, crabs [100]. Subadult and adult Kemp’s ridleys primarily feed on crabs, tunicates, bivalves, gastropods, seagrass, algae, ascidians, comb jellies, and fishes [100,101]. Both loggerheads and Kemp’s ridleys may ingest brevetoxins by feeding on a variety of marine invertebrates and secondary consumers. While mature green turtles are primarily herbivorous, immature green turtles may, in addition to feeding on seagrass and algae, occasionally feed on tunicates, constituting an additional source of brevetoxin exposure to juveniles [102].

Although fish and marine invertebrates pose a significant route of exposure to more carnivorous sea turtles, primarily herbivorous adult green turtles may also be exposed to brevetoxins through their diet. Seagrass may act as a constant source of brevetoxin exposure, as brevetoxin metabolites were found to persist for up to eight months in seagrass communities after a bloom had subsided [92]. Moreover, seagrass has been implicated in mortality events in Florida manatees (*Trichechus manatus latirostris*) and likely constitutes a major route of exposure for green turtles [17]. Sea turtle stranding demographics depict relatively few green turtles during red tide events, which could be a result of lower dietary exposure through seagrass than other sea turtle species through their respective diets [15]. 

Dietary preference may, in part, explain why adult and large immature loggerheads and Kemp’s ridleys strand more frequently than green turtles during *K. brevis* blooms [15]. Another factor related to dietary exposure may be the spatial distribution of sea turtle species. Green turtles may prefer coastal inland waters, richer in seagrass, whereas Kemp’s ridleys and loggerheads prefer coastal inland and shelf waters [15,103]. *K. brevis* blooms are more frequent in shelf waters than in coastal inland waters, meaning Kemp’s ridleys and loggerheads may be exposed more frequently and more severely to *K. brevis* HABs than green turtles [12]. Differences in stranding demographics may also occur as a result of the movement of blooms to nearshore waters, which may result in the increased mortality of juvenile and subadult turtles [40]. Furthermore, foraging site fidelity has been observed in loggerheads, green turtles, and Kemp’s ridleys, which may result in recurrent exposure if toxin burdens at a specific foraging site are elevated [104,105,106]. Satellite telemetry data have, however, suggested that Kemp’s ridleys, and perhaps other sea turtles, display avoidance behavior to harmful algae, although more research is required to further support these observations [107].

### 3.2. Cutaneous Absorption

Similar to other organic, lipophilic contaminants that have been detected in dermis samples from green turtles and hawksbill sea turtles (*Eretmochelys imbricata*) [108], cutaneous absorption may constitute an additional route of brevetoxin exposure in sea turtles. While the absorption of toxins through the buccal mucosa is likely greater than through the dermis, the penetration of brevetoxins through dermal tissue has been demonstrated in vitro in human, rhesus macaque (*Macaca mulatta*), guinea pig (*Cavia porcellus*), and pig (*Sus domesticus*) dermal tissues [109,110,111]. Cutaneous absorption may be of importance to sea turtles, as they are continuously exposed to brevetoxins during HAB events. Future research may focus on identifying the significance of this exposure route to brevetoxins in sea turtles.

### 3.3. Aerogenic Exposure

*K. brevis* is unarmored; therefore, the organism is easily destroyed by wave action along beaches, causing intracellular toxins to be released into the water column. Furthermore, brevetoxins and *K. brevis* fragments may attach to aerosolized droplets and salt particles, which can be carried along vast distances over the air [112]. As a result, weather conditions may impact the dispersal of toxins and increase an HAB’s area of impact. It has been determined that PbTx-3 is the primary compound present in aerosols, followed by PbTx-2. No PbTx-1 has been detected in aerosols [13]. PbTx-3 is reported to be primarily responsible for respiratory irritation and bronchoconstriction in a guinea pig model [113] and is likely the primary cause of human airway irritation following exposure to aerosolized brevetoxins.

Toxicity following respiratory exposure to brevetoxins has also been reported in turtles. Intratracheal exposure of as little as 4.68 mg PbTx-3/kg caused reduced activity following experimental exposure of red-eared sliders (*Trachemys scripta*) [114]. Neurological symptoms have been observed within two to five minutes following intratracheal toxin instillation at a low PbTx-3 dosage (10.53 mg/kg) [115]. In comparison, following oral exposure, the onset of neurological symptoms occurred after roughly 30 min with a dosage of 33.48 mg/kg PbTx-3 [115].

Sea turtles may be particularly susceptible to respiratory brevetoxin exposure. Sea turtles display a pre-dive breathing pattern consisting of rapid inhalation with a large tidal volume that approaches their vital capacity. This may result in an increased intake of aerosolized brevetoxins [116]. Moreover, loggerhead sea turtles may be particularly susceptible to the effect of aerosolized brevetoxins. The efficacy of diphenhydramine treatment in reducing conjunctival edema in loggerheads implies that aerogenic exposure to brevetoxins may cause hypersensitivity reactions in this species, adding to morbidity [117].

### 3.4. Vertical Transfer

Research on elasmobranchs suggests that the vertical transfer of brevetoxins occurs and that brevetoxin exposure during the egg development stage is significant in viviparous species [118]. Similarly, the vertical transfer of brevetoxins may constitute a route of exposure to hatchling sea turtles. Brevetoxins, as lipophilic compounds, accumulate in tissues and may be released as lipid stores are metabolized during periods of vitellogenesis and egg-laying [119]. The vertical transfer of toxins has been observed in loggerheads and green turtles [120]. Liver concentrations of dead-in-nest hatchlings significantly correlated with plasma brevetoxin concentrations of nesting females [120]. While hatchling liver concentrations were observed to be lower than the liver concentrations reported in dead stranded loggerheads, green turtles, and Kemp’s ridleys [38,40,120], liver brevetoxin concentrations of hatchling sea turtles overlapped with those reported in live stranded loggerhead turtles that later died while undergoing rehabilitation, indicating that these brevetoxin concentrations may negatively impact the health of hatchling sea turtles [40,120].

For mature sea turtles, vertical transfer poses a mechanism to offload toxins [121]. Lower plasma brevetoxin concentrations were observed in nesting loggerheads compared to other loggerheads and Kemp’s ridley turtles sampled during *K. brevis* HABs [40,103,116,120]. The sampling of the female loggerheads occurred months after the record of the last bloom in the area. Low plasma brevetoxin concentrations are, therefore, likely the result of toxins being released during vitellogenesis and from adipose tissue during the period of reduced food intake leading up to nesting [120]. It is likely that plasma brevetoxin concentrations remain constant during periods of high activity and low food intake, which may already occur during migration to the nesting beach [121]. Furthermore, it is unlikely that brevetoxin concentrations in nesting loggerheads were the result of primary exposure, as no parent congeners PbTx-1 and PbTx-2 were observed, and only low concentrations of PbTx-3 were reported, suggesting the presence of metabolites in the plasma of nesting females [120]. Parent congeners were also observed to be low or absent on LC-MS/MS analysis in eggs and hatchling tissues, indicating that brevetoxin metabolites are predominantly passed on to offspring [120].

The allocation of nutrients and energy toward addressing toxic stress caused by sublethal brevetoxin exposure in hatchling sea turtles may impact their developmental success. Furthermore, it has been observed that hatching and emergence success decreased with increasing yolk sac brevetoxin concentrations [120]. The presumed impact of brevetoxins on reproductive success further corroborates that the effects of toxins may extend beyond what can be seen during HAB-associated stranding events. 

## 4. Pathophysiology

### 4.1. Central Nervous System

All brevetoxin compounds bind to neurotoxin receptor site five associated with domain VI of the α-subunit of open voltage-gated sodium channels (VGSCs) [14]. Moreover, the high-affinity binding of brevetoxin prevents VGSC inactivation, which causes cellular depolarization and the release of glutamate, which induces secondary activation of *N*-methyl-d-aspartate (NMDA) and α-amino-3-hydroxy-5-methyl-4-isoxazolepropionic acid receptor (AMPA) receptors [122]. The subsequent Ca^2+^ influx triggers a cascade of events leading to oxidative stress, DNA damage, modified cell proliferation, and cellular apoptosis [123,124]. The cellular depolarization that results from brevetoxin exposure may directly affect skeletal and cardiac systems and can cause neurological deficits [125,126]. 

A freshwater turtle model demonstrated resistance of turtle neurons to the effects of PbTx-3 compared to mammalian studies [90,114]. Anoxia causes mild depolarization in turtle neurons, inhibits the release of excitotoxic neurotransmitters, and decreases the activity of ion channels and NMDA receptors [127,128]. This is thought to be a mechanism to maintain ion gradients in the face of decreased ATP availability during anoxia [90]. The ability to reduce NMDA receptor activity may, in part, explain the resistance of turtle neurons to brevetoxin-induced glutamate toxicity [90]. When extrapolating these data to sea turtles, however, it must be kept in mind that these freshwater turtles are being exposed to brevetoxins for the first time, whereas sea turtles are likely exposed to brevetoxins on a regular basis. Differences in physiology between freshwater and sea turtles could also influence the exact neurological pathophysiology.

### 4.2. Immune Effects

Innate immunity is important in turtles, as their low metabolic rate is associated with a slowly developing acquired immune response of low magnitude [129]. Circulating lysozyme is a measure of innate immunity and a marker for pro-inflammatory responses and has been reported to play a role in oxidative stress [116,130]. Plasma lysozyme activity positively correlated with brevetoxin concentrations in stranded loggerheads brought to rehabilitation, which suggests that brevetoxins may have an immunomodulating impact [116]. In contrast, a reduced plasma lysozyme activity was observed one hour after oral exposure to PbTx-3 in freshwater turtles, which may be indicative of potential immunosuppression [115]. It is possible that the exposure of wild sea turtles to a variety of brevetoxin congeners for more extended periods contributes to this difference. However, it may also be that the lysozyme activity reported in stranded sea turtles was increased as a result of other debilitating conditions that may or may not have been a result of brevetoxin exposure. In freshwater turtles, a significant decrease in plasma lysozyme activity was only observed one hour after oral exposure and not at subsequent points in time. It may be that the increased plasma lysozyme activity observed in stranded loggerheads was a result of upregulation following longer-term brevetoxin exposure.

The immunomodulatory effects of brevetoxins may also extend to the adaptive immune system. A positive correlation between brevetoxin and γ-globulin concentration was observed in green turtles and nesting loggerheads, with γ-globulin concentrations exceeding reference ranges in green turtles [25,120]. Although not evaluated in sea turtles, significant effects on T- and B-lymphocyte proliferation were observed following four weeks of oral exposure of freshwater turtles to PbTx-3 [115]. The immune function may be further impacted by brevetoxin-induced alterations in microtubule formation and antigen presentation, as has been suggested by gene expression analysis in rescued loggerhead peripheral blood lymphocytes and in a freshwater turtle model [115,116].

The immune effects of brevetoxins are increasingly understood across species [19,131,132,133,134,135]. While the exact impact of brevetoxins on the immune system of sea turtles remains unclear, an influence on various aspects of the immune system is highly suspected and should be investigated further. An altered immune status may have far-reaching consequences for sea turtles, as they may become more susceptible to opportunistic infections or the reactivation of latent infections [136].

### 4.3. Inflammation

It has been observed that plasma α-globulin concentrations increase in response to infections and inflammatory processes. Increased α-globulin concentrations were observed in free-ranging Kemp’s ridleys without clinical signs after a *K. brevis* bloom [25], and insignificant increases in α-globulin concentrations have been reported in a small group of free-ranging Kemp’s ridleys and in a freshwater turtle model [103,115]. No correlation was found between plasma β- or γ-globulin concentrations and brevetoxin exposure in free-ranging Kemp’s ridleys and in experimentally challenged red-eared sliders [103,115]. Overall, the plasma electrophoresis profile in the freshwater turtle model yielded no significant changes that concurred with findings in wild sea turtles [115]. Physiological differences between animal species and exposure to different congeners and concentrations of brevetoxins may account, in part, for these differences. Overall, no strong evidence currently suggests that inflammation occurs as a major response to brevetoxin exposure in turtles, although studies using other markers, such as serum amyloid A (SAA), may still further our understanding of the effect of brevetoxins on inflammatory processes [115].

### 4.4. Oxidative Stress

Brevetoxins, particularly PbTx-2, inhibit thioredoxin reductase activity in mammals, which provides a potential mechanism resulting in oxidative stress [137]. This mechanism is supported in turtles by gene expression analysis in rescued loggerheads, in which peripheral blood lymphocytes that were exposed to PbTx-2 in vitro revealed an upregulation of genes with roles in oxidative stress, such as NADH-ubiquinone oxidoreductase, superoxide dismutase (SOD), thioredoxin, and ferritin heavy chain [116]. Following the exposure of red-eared sliders to PbTx-3, similar increases in SOD and thioredoxin were observed, as well as increases in glutathione-S-transferase (GST) and heat shock proteins [115]. Increased SOD activity has been reported in stranded Kemp’s ridleys with higher plasma brevetoxin concentrations following brevetoxin exposure but did not correlate with brevetoxin exposure in clinically healthy wild Kemp’s ridleys or in nesting loggerheads [25,103,120]. The effects of brevetoxins on the expression of genes associated with oxidative stress may not have been observed in wild Kemp’s ridleys or nesting loggerheads as a result of the small sample size, exposure to lower brevetoxin concentrations, or the fact that only the SOD gene was evaluated. In experimental PbTx-3 exposure of freshwater turtles, elevated SOD was observed following intratracheal exposure but not following oral exposure. This difference could be attributed to the hepatic first-pass metabolism of PbTx-3 following oral exposure [115]. It may also play a role that only the PbTx-3 congener was used in this freshwater turtle model, whereas the PbTx-2 has been associated with the inhibition of thioredoxin reductase activity. This would, however, not explain why elevated SOD activity was observed following intratracheal exposure to PbTx-3 in freshwater turtles [115,137]. 

Analysis of peripheral blood lymphocytes collected from *K. brevis* HAB-rescued sea turtles indicated that genes involved in oxidative stress and xenobiotic metabolism were upregulated, indicating a significant alteration in normal cellular functioning following brevetoxin exposure [116]. Brevetoxin exposure may be associated with the formation of reactive oxygen species and could result in the upregulation of genes associated with oxidative stress. Future research may focus on assessing the effect of brevetoxins on cellular function in sea turtles using larger sample sizes, assessing a relationship with differing brevetoxin concentrations, and through the analysis of multiple genes associated with oxidative stress.

### 4.5. Pharmacokinetics

#### 4.5.1. Tissue Distribution

Brevetoxins have been observed to be distributed to all organ systems in freshwater turtles, with the highest concentrations being observed in the liver, kidneys, intestines, bile, feces, and urine [114,138]. 

Brevetoxins are transported via the blood plasma, bound to high-density lipoproteins (HDL), and can, therefore, be demonstrated in blood samples [139]. Interestingly, plasma brevetoxin concentrations in free-ranging Kemp’s ridleys have been observed to be similar to those measured during stranding events [103]. It has been proposed that this difference may be due to anorexia and continuation of toxin clearance leading up to stranding, which reduces plasma brevetoxin concentrations of stranded sea turtles [103]. Plasma and whole blood PbTx-3 concentrations in live stranded green turtles appear to be lower than those found in loggerheads and Kemp’s ridleys [40]. This difference may, in part, be a result of dietary preference [25,41]. Plasma brevetoxin concentrations in smaller sea turtles, as measured by both mass and carapace length, have been observed to be higher than in larger individuals [40]. It has been proposed that a growth dilution effect may occur. As tissues grow, a dilution of brevetoxins may occur as long as environmental brevetoxin exposure is stable [103]. Another survey, however, observed no significant difference in carapace length between sea turtles stranded during HAB events and sea turtles stranded turtles during HAB absence [15].

Kidney brevetoxin concentrations appear to be highly variable in all sea turtle species (Table 1). The highest mean concentrations have been observed in dead stranded Kemp’s ridleys (151 ng PbTx-3 eq/g), but the largest range was reported in loggerheads, with values reaching 408 ng PbTx-3 eq/g [40]. Data on brevetoxin concentrations in urine samples are scarce, but the results from live stranded loggerheads and Kemp’s ridleys suggest urinary brevetoxin concentrations are low [40]. These low urine brevetoxin concentrations and increased concentrations in the bile and feces over time suggest that hepatic excretion is the main route of toxin clearance [114].

Although liver brevetoxin concentrations may show large degrees of variation between individuals, it appears that the differences between turtle species are small (Table 1). Mean liver toxin concentrations in stranded loggerheads, green turtles, and Kemp’s ridleys were similar to each other and to those reported in experimentally challenged freshwater turtles [40,114]. The largest degree of variation was reported in dead stranded Kemp’s ridley sea turtles, where liver brevetoxin concentrations were reported to range from <1 to 1006 ng PbTx-3 eq/g. Data suggest that liver brevetoxin concentrations are lowest in live stranded turtles that die after entering rehabilitation, but this difference is likely a result of the continued excretion of brevetoxins in rescue centers prior to death [40].

High brevetoxin concentrations have been reported in feces (Table 1). This could be explained by the excretion of brevetoxins through the bile, but concentrations may also largely consist of toxins passing through the GI tract unabsorbed. This is supported by the trend that the highest brevetoxin concentrations were determined in stomach content samples (Table 1). Although lower plasma brevetoxin concentrations in green turtles have previously been attributed to dietary preference, the highest median brevetoxin concentration was reported in the stomach contents of dead stranded green sea turtles [40]. The lowest mean concentrations, however, were also reported in dead stranded green turtles in a later study [3]. Overall, the range of stomach content brevetoxin concentrations is smaller in dead stranded green turtles than in dead stranded Kemp’s ridleys and live stranded loggerheads, but large ranges have been observed in all species (Table 1). 

#### 4.5.2. Brevetoxin Clearance

The clearance of brevetoxins from the body is vital for the recovery of afflicted sea turtles. In experimentally exposed freshwater turtles, neurological symptoms were most severe at one to six hours after PbTx-3 exposure and diminished over 24 h. However, lethargy persisted for at least two to four weeks, suggesting that the full clearance of toxins is a slow process [114]. Furthermore, it has been suggested that an impaired health status may reduce the ability of turtles to clear toxins, which may trigger a cycle of toxin-induced alterations to the immune function and neural and muscular activity that result in a reduced body condition, which, in turn, reduces the animals’ ability to excrete toxins [114]. This has been observed in wild sea turtles, as the majority of brevetoxin-afflicted sea turtles that survived rehabilitation eliminated over 80% of toxins from their body by day 20, whereas animals that died during rehabilitation only eliminated 40–60% of toxins by day 20 [40]. Interestingly, in some cases, increases in plasma brevetoxin concentrations were observed at various time points in rescued sea turtles compared to plasma concentrations at the time of admission. These turtles were provided with fluid therapy, which was expected to dilute plasma brevetoxins. The observed increases in plasma brevetoxin concentrations may be attributed to the continued digestion of contaminated prey and reabsorption of brevetoxins from the gut [40,41]. Clearance rates may also depend on the sea turtle species involved. Naturally exposed loggerheads cleared brevetoxin concentrations after 5–80 days, whereas green turtles and Kemp’s ridleys managed to do so in 2–15 days [40]. These data suggest that brevetoxins may persist in sea turtles for prolonged periods, as has been corroborated by the finding of similar brevetoxin concentrations in the blood of Kemp’s ridley turtles several months after dissipation of the bloom [103], as well as the observation of brevetoxins in the plasma of nesting female loggerheads several months after the occurrence of a *K. brevis* bloom [120]. Although continued dietary exposure may have played a role in these studies, it is likely that these plasma concentrations also, partially, resulted from the slow release of tissue-accumulated brevetoxins. The sequestration of brevetoxins in adipose tissue and its subsequent slow release may result in health effects long after an HAB has subsided, particularly during periods in which fat stores are metabolized, such as during nesting [120].

## 5. Clinical Assessment

### 5.1. Clinical Symptoms

Brevetoxin-affected sea turtles generally present with neurological symptoms, which may include loss of muscle coordination, circling behavior, head bobbing, inability to submerge, muscle twitching, and myoclonus. More severe symptoms may include generalized lethargy, partial-to-complete paralysis, and unresponsiveness [40]. Furthermore, seizures may occur in severe cases [140]. While moderate symptoms may impair the animals’ ability to forage and evade predators, generalized lethargy and partial paralysis may compromise the animals’ ability to swim and breathe [41]. This may result in an increase in entanglements and boat strikes, which has been reported in stranded brevetoxin-afflicted sea turtles [42]. 

Whereas the aforementioned symptoms may occur in all sea turtle species, symptoms appear to be more severe in loggerheads. Moreover, additional symptoms reported in this species include generalized edema, conjunctival edema, cloacal prolapse in adult females and subadults of both sexes, and penile prolapse in adult males [117]. Similarly, penile prolapse and edema have been observed in experimentally exposed *T. scripta* [114]. It is thought that the increased severity of symptoms in loggerheads may be a result of differences in toxin distribution, dietary preferences, delayed gut passage, or the presence of blood flukes affecting blood circulation in various organs [40,141,142]. More research is required to further understand the apparent increased severity of clinical disease in loggerheads.

Brevetoxin-afflicted sea turtles may present with a variety of secondary conditions. Frequently reported conditions include bacterial infection, dehydration, hypoglycemia, gastrointestinal ailments, anemia, malnutrition, spirorchiid infection, and metabolic acidosis [41]. Fungal infections, in particular fungal pneumonia, may also be present in affected sea turtles [140]. Furthermore, it is possible that sublethal exposure to brevetoxins predisposes sea turtles to accidental death by reducing their ability to evade boats, predation, or fishing lines and may increase overall morbidity during cold weather snaps [38].

### 5.2. Diagnostic Options

A significant correlation between plasma and liver brevetoxin concentrations has been observed. As a result, plasma brevetoxin concentrations may provide a useful estimate of brevetoxin exposure without the need for the collection of liver samples [115]. Ultracentrifugation of blood samples may not be necessary, as only a small but insignificant difference in brevetoxin concentrations has been observed between lipemic plasma samples that were analyzed native versus those that were subjected to lipid extraction by ultracentrifugation [41]. 

A competitive enzyme-linked immunosorbent assay (ELISA) may be used to determine brevetoxin concentrations in tissues and plasma. This assay detects brevetoxin congeners with the dominant (80%) B-type backbone (PbTx-2, 3, 5, 6, 8, 9). Congeners with the A-type backbone (PbTx-1, 7, 10) are recognized at reduced affinity [40,94]. The results from the ELISA are the sum of the detected congeners, which are frequently simplified and reported as ng PbTx-3/mL [143]. In order to determine the presence of specific congeners, high-performance liquid chromatography with mass spectrometry (LC-MS) may be performed [40,94].

Brevetoxins are highly stable compounds and are still being detectable in 60–80-year-old sediments [144]. Brevetoxins were observed to be stable in the presence of 50 ppm chlorine at temperatures exceeding 300 °C and at a pH range of 2–10 [145]. Brevetoxins were observed to be stable in plasma samples stored at −20 °C for at least one month and were still measurable after 32 months in blood samples stored on cellulose blood collection cards [146,147]. Freeze-thawing negatively impacts brevetoxin stability in plasma samples [146].

Histopathology has revealed no definitive pathological changes associated with brevetoxicosis in wild sea turtles or freshwater turtle models [40,114,138]. Similarly, no lesions have been associated with brevetoxicosis on gross necropsy. Malnutrition, spirorchiid infection, and pneumonia have been reported in sea turtles that died from brevetoxicosis, but these issues are likely secondary and are not specific to brevetoxicosis [40]. Weakness or paralysis may cause aspiration pneumonia and malnutrition, manifested as hepatic and serous fat atrophy, which may result from reduced foraging ability. As no postmortem lesions are specific to brevetoxicosis, the postmortem diagnosis of dead stranded sea turtles or sea turtles that die during rehabilitation relies on demonstrating the presence of brevetoxins in tissues. It has been reported that these toxins were detected in at least one sample from 98% (n = 43) of dead stranded sea turtles and 93% (n = 56) of live stranded turtles using ELISA, although sensitivity varied per sample type [40]. Concentrations may have to be considered qualitatively in older carcasses, as information on the stability of brevetoxin congeners in decomposing tissues and the role of cross-contamination from scavenging is lacking [3].

## 6. Therapeutic Options

### 6.1. Intravenous Lipid Emulsion Treatment

Intravenous lipid emulsion (ILE) treatment is the current therapy of choice for brevetoxin-afflicted sea turtles. ILE has been used in the treatment of various acute intoxications of lipid-soluble toxins and has been observed to be effective in sea turtles [41,148]. ILE is thought to work by drawing out toxins from cells or preventing toxins from reaching target tissues by creating a high volume of lipids in the extracellular compartment, a so-called lipid sink [149]. However, recent research suggests that ILE works by scavenging local lipophilic compounds from high blood flow organs, such as the brain, and redistributing them to muscles for storage and the liver for detoxification [150]. Future research analyzing muscle samples following ILE therapy may shed more light on the mechanism underlying the effect of ILE in sea turtles with brevetoxicosis. 

In a freshwater turtle model, the administration of 100 mg/kg ILE 30 min prior to brevetoxin (as PbTx-3) exposure was successful in preventing symptoms, and post-exposure treatment with the same dosage resulted in a significant and rapid reduction in clinical symptoms. Lower brevetoxin clearance rates and slower resolution of clinical symptoms were observed in freshwater turtles treated with 50 mg/kg ILE. ILE is fast-acting in turtles, as brevetoxin concentrations in the kidneys, liver, brain, and heart decreased significantly within 1 h after ILE delivery. PbTx-3 concentrations in kidneys and liver increased after 24 h after higher dosages of ILE compared to lower dosage ILE-treated animals, suggesting increased transport to excretory systems following ILE treatment [138].

In sea turtles, ILE was observed to reduce nearly all symptoms of brevetoxicosis within 24–48 h when given at a dosage of 25 mg/kg, significantly faster than standard and supportive care [41]. Concurrently, plasma brevetoxin concentrations declined at a faster rate in loggerheads and Kemp’s ridleys using ILE compared to supportive care. A 50% reduction in plasma brevetoxin concentrations was observed 115 and 71 h faster in ILE-treated loggerheads and Kemp’s ridleys, respectively, in comparison to those that received standard and supportive care [41]. Moreover, full clearance of plasma brevetoxins was estimated to occur 696 h and 275 h faster in ILE-treated loggerheads and Kemp’s ridleys, respectively, in comparison to those receiving standard and supportive care [41].

The reported survival rate following ILE (94%) is significantly higher than the survival rate of turtles receiving supportive care and/or dehydration therapy (47%) [41]. A higher dosage, such as 50–100 mg/kg, has been reported to be safe in freshwater turtles and has been used safely in a limited number of sea turtles. This increased dosage may further increase the efficacy of ILE treatment in sea turtles [41,138]. 

### 6.2. Osmotic Treatment

A treatment using the diuretic mannitol (0.5 mg/kg i.v.) has been attempted, but no clinical response was observed. This may have been a result of a low dosage and a small treatment group. Further research is required to investigate the efficacy of higher dosage mannitol in the treatment of brevetoxicosis [117].

Dehydration treatment resulted in a marked increase in successful rehabilitation and release, from 12% (2 of 17) without receiving dehydration therapy to 71% (5 of 7) with this therapy. The treatment consists of the administration of furosemide (5 mg/kg i.m. q24h) whilst simultaneously withholding all fluids for two to three days. Obvious reductions in neurological symptoms were observed within one to three days. The mechanism underlying this therapy is unknown. Although the response to dehydration treatment suggests central nervous system edema, no histopathologic evidence supports this theory. Moreover, the statistical significance of these observations is limited, as only seven sea turtles received dehydration treatment [117]. It has also been proposed that a direct interaction between furosemide and sodium channels in the central nervous system may underlie the efficacy [117]. Further research is needed to better understand the mechanism by which furosemide treatment reduces brevetoxin-associated symptoms. If furosemide acts through direct interaction with sodium channels, there may be no need for concurrent dehydration, which opens the possibility of combining furosemide treatment with ILE treatment.

### 6.3. Standard and Supportive Care

Initially, sea turtles were treated by placing the animals in a toxin-free environment with their heads propped up above the water to prevent drowning while the toxins cleared [40,117]. Supportive treatment mainly consisted of gavage feeding and subcutaneous fluid therapy. The success rate of this treatment was low as only 36% (14 of 39) of loggerheads, 29% (two of seven) of green turtles, and 83% (five of six) of Kemp’s ridleys were successfully released using this treatment plan [40]. In another study, the success rate in green and Kemp’s ridley sea turtles was 100% (n = 5) after 1–1.5 months of rehabilitation, although the treatment was ineffective in loggerheads [117]. A more aggressive treatment was attempted in loggerheads, consisting of the oral administration of activated charcoal–kaolin suspension, freshwater hydration therapy, and metoclopramide to stimulate gastrointestinal motility. This aggressive treatment regimen, however, resulted in a low release rate of 12% (2 of 17) of loggerheads [117]. 

The reported reduced efficacy of supportive treatment as opposed to dehydration and ILE treatment should not be used as a reason to withhold supportive care. The aforementioned supportive therapeutic options should be used in conjunction with more advanced treatments if possible. Total parenteral nutrition (TPN) has been used in sea turtles suffering from chronic debilitation syndrome [151]. Similarly, the administration of TPN twice daily has been observed to be beneficial for chronically debilitated sea turtles affected by brevetoxicosis and may be used in conjunction with ILE treatment [140].

### 6.4. Cholestyramine Treatment

It has been hypothesized that the enterohepatic recirculation of brevetoxins may impair the biliary clearance of brevetoxins in sea turtles, prolonging morbidity [40]. Cholestyramine, an anion exchange resin, strongly binds to bile salts and prevents reabsorption and has been successfully used to increase the biliary clearance of microcystin intoxication in mammals [152]. Oral dosages of 20 mg/kg and 50 mg/kg were evaluated in a freshwater turtle model, but no significant changes in biliary brevetoxin concentrations were observed after 24 h compared to untreated PbTx-3-exposed control animals. Although not statistically significant, a decrease in brevetoxins in the kidneys, liver, feces, heart, lungs, brain, and fat was observed. The lack of significance may be a result of high variability in brevetoxin concentrations in samples of PbTx-3-exposed control animals, which could be controlled by repeated dosing of brevetoxin over a four-week period. This would result in higher tissue and plasma brevetoxin concentrations, thus providing a more robust control measure. It may also be possible that the cholestyramine dosage was insufficient, and higher dosages may be investigated in the future [138]. 

### 6.5. Other Treatment Options

Brevenal is a VGSC antagonist produced by *K. brevis* and naturally occurs in low concentrations during *K. brevis* blooms [13,153]. Brevenal has been demonstrated to reduce brevetoxin-associated cell death in vitro and was observed to be non-toxic at concentrations of 1 mg/mL in a fish bioassay. Moreover, brevenal significantly delayed the mortality of fish upon exposure to 1 mg/mL PbTx-2. Brevenal and the brevenal-related derivative, brevenal acetal, have the potential to be used as therapeutic agents for the treatment of brevetoxicosis, but further research is required to assess the efficacy and safety of this treatment [153].

Diphenhydramine was observed to rapidly reduce conjunctival edema and, as a result, may prevent corneal ulceration in sea turtles when given at 2 mg/kg i.m. q24h. This treatment appears to be particularly useful in reducing conjunctival edema but shows little efficacy in reducing other symptoms. It has been proposed that conjunctival edema occurs as a hypersensitivity reaction and as such, may have a different underlying pathophysiology to other symptoms [117]. This is corroborated by the fact that ILE treatment appears to be ineffective against conjunctival swelling [41].

A complete overview of treatment options is provided in Table 2.

### 6.6. Treatment of Secondary Conditions

Minor cloacal prolapse may be treated with topical antibiotic ointment to control pressure necrosis and reduce the risk of infection. Penile prolapse may be manually reduced and loosely bandaged around the tail to prevent the reoccurrence of the prolapse without obstructing waste elimination. The wrap may be removed when the penis is no longer prolapsed, usually within a few days [117]. Alternatively, if the sea turtle’s condition allows it, returning the animal to the water is likely to resolve penile prolapse on its own [155].

Corneal ulcers may develop following the loss of ocular reflexes. When no palpebral reflex is present, the sea turtles’ eyes should be lubricated frequently to prevent infection and ulceration. In cases where corneal ulcers have been confirmed using fluorescein staining, medication of the eyes with antibiotic ointment should be considered [140]. Additionally, surgical tarsorrhaphy may be required in animals with deep corneal ulcers [117]. 

Concurrent spirorchiidiasis may be treated with praziquantel (25 mg/kg p.o. t.i.d. for 1 day) [117].

## 7. Future Perspectives

Despite significant advancements in the past decade regarding our comprehension and management of brevetoxicosis in sea turtles, there remains ample room for research in this area. Gaining a deeper understanding of the contributing factors behind the occurrence of *K. brevis* HABs could potentially enable us to minimize their impact through the implementation of mitigation strategies and prompt response to stranding events. More effective monitoring strategies may also be developed. Due to the high degrees of foraging site fidelity displayed by sea turtles [104,105,106], it should be worthwhile to monitor brevetoxin exposure in high-density foraging areas.

Future research may also focus on the relationship between brevetoxin exposure and other threats to sea turtles, such as fibropapillomatosis, susceptibility to ghost gear entanglement or boat collision, and other toxicants [24,25]. The sampling of long-term brevetoxin exposure in stranded sea turtles and relating these observations to determined tumor scores may shed light on a potential relationship between fibropapillomatosis and brevetoxin exposure [24]. To further understand the long-term impact of brevetoxicosis, the separate analysis of brevetoxins in egg yolk and albumen will provide more information on the vertical transfer of brevetoxins, as the toxins in the albumen will also be consumed and utilized by the embryo [120]. Furthermore, due to a global increase in HABs, the synergistic effects of multiple marine microalgal toxins should be investigated.

By furthering our understanding of brevetoxin pharmacokinetics, we may better understand the broad pathophysiology of brevetoxicosis, as well as its impact on the immune system. Moreover, research on pharmacokinetics should help further our understanding of the impact of brevetoxins on hatchling sea turtles [120].

Current treatments may be further refined by finetuning the effective dosage of ILE treatment. Moreover, the analysis of muscle samples following ILE therapy may shed more light on the mechanism underlying the efficacy of ILE, potentially guiding future research. In addition, investigation into the application of ILE for water-soluble toxins will help elucidate ILE treatment mechanisms.

Novel treatment options, such as the application of brevenal, may be further investigated. Clinical outcomes could potentially be improved by employing a combination of treatment options. However, due to the limited understanding of the mechanisms underlying treatment options, it is currently challenging to accurately predict which treatment options would synergistically work together.

## 8. Conclusions

This article provides a review of the impact of *K. brevis* HABs on sea turtles. The current understanding of the toxicopathological mechanisms underlying brevetoxicosis, as well as the impact of brevetoxins on the overall health of sea turtles, is discussed. Diagnostic approaches in both dead and live stranded turtles should focus on demonstrating brevetoxins in samples. Considerable research has explored therapeutic interventions like supportive care, dehydration treatment, and ILE therapy. However, knowledge gaps remain around the impact of brevetoxins on sea turtle health in a broad sense, such as reproductive success and the possibility of increased susceptibility to other threats. Continued research in these areas, including the optimization of treatment protocols, will help mitigate threats to already endangered sea turtle species. As harmful algal blooms are anticipated to change with climate change, future research is vital to safeguard sea turtle populations in the face of this growing challenge.

## Figures and Tables

**Table 1:** Brevetoxin concentrations (ng PbTx-3/g or mL) reported in the literature in different samples under a variety of conditions in loggerhead (*Caretta caretta*), green (*Chelonia mydas*), and Kemp’s ridley (*Lepidochelys kempii*) sea turtles.

		Plasma			Whole Blood			Lung			Kidney			Liver			Bile			Stomach Contents			Feces			Urine			
Species	Animal Status	Samples (n)	Mean	Range	Samples (n)	Mean	Range	Samples (n)	Mean	Range	Samples (n)	Mean	Range	Samples (n)	Mean	Range	Samples (n)	Mean	Range	Samples (n)	Mean	Range	Samples (n)	Mean	Range	Samples (n)	Mean	Range	Source
*C. caretta*	Dead Stranded							15	63	<1–438	16	63	<1–408	17	131	<1–683	3	23	<1–50	15	142	<1–971	13	402	<1–3139				[40]
*C. caretta*	Live Stranded	34	32	<1–107	33	25	<1–81	13	11	<1–32	23	17	<1–138	22	43	<1–470	20	50	<1–427	15	865	<1–11,804	8	8398	30–61,078	1	1.7		[40]
*C. caretta*	Live Stranded	9	32.8	1.6–64.6																									[41]
*C. caretta*	Live Stranded	9	68	34.0–89.0																									[116]
*C. caretta*	Nesting	48	9.1	2.1–26.7																									[120]
*C. mydas*	Dead Stranded													6	20	5.6–40.9													[38]
*C. mydas*	Dead Stranded							5	144	<1–396	6	90	<1–200	6	186	<1–345	1	220		6	677	<1–1312	3	968	<1–1126				[40]
*C. mydas*	Dead Stranded										5	12.8	2–34	5	24.4	6–75				5	56.7	7–100							[3]
*C. mydas*	Live Stranded	6	1.2	1–4	6	1.6	<1–4										4	14	<1–23				5	102	<1–382				[40]
*C. mydas*	Live Stranded	4	10	4.9–15.3																									[41]
*C. mydas*	Wild Caught	8	1.7	<1.0–5.2																									[25]
*C. mydas*	Wild Caught	21	< 75																										[26]
*L. kempii*	Dead Stranded							19	131	<1–247	20	151	<1–348	20	297	<1–1006				19	578	<1–3522	17	545	<1–1832				[40]
*L. kempii*	Dead Stranded										2	105	67–143	7	215.1	25–372				7	360.3	9–1687							[3]
*L. kempii*	Live Stranded	5	63	<1–82	5	36	<1–82																4	679	390–877	1	7.4		[40]
*L. kempii*	Live Stranded	5	41.2	5.0–93.4																									[41]
*L. kempii*	Wild Caught	9	22.6	13.0–33.8																									[103]
*L. kempii*	Wild Caught	21	2.6	<1.0–8.6																									[25]

**Table 2:** Overview of treatment results in loggerhead, green, and Kemp’s ridley sea turtles reported in the literature.

References	Treatment	Species	Turtles Released/Total Turtles Admitted with PbTx-Positive ELISA (%)	Time until Resolution of Neurological Signs (Days)	Time until Plasma PbTx-3 Concentrations Reached < 10 ng PbTx-3/mL (Days)	Time in Rehabilitation (Days) ^	Comments
[41]	ILE (25 mg/kg)	*C. caretta*	9/9 (100%)	1–2	7	14–62	
[41]	ILE (25 mg/kg) ^#^	*L. kempii*	4/5 (80%)	1–2	7	14–26	
[41]	ILE (25 mg/kg)	*C. mydas*	4/4 (100%)	1–2	<1	14–19	
[41]	ILE (25 mg/kg) ^#^	*C. caretta*, *L. kempii*, *C. mydas*	17/18 (94%)	1–2	<1–7	14–62	Combined data across sea turtle species.
[138]	ILE (50–100 mg/kg)	*T. scripta*	N/A	N/A	N/A	N/A	
[117]	Dehydration therapy (Furosemide 5 mg/kg IM)	*C. caretta*	5/7 (71%)	N/A ^~^	N/A	41–87	
[117]	Dehydration therapy (Mannitol 0.5 mg/kg IV)	*C. caretta*	0/2 (0%)	N/A	N/A	N/A	
[154]	Supportive care	*C. caretta*	2/4 (50%)	>7	N/A	14–26	
[40]	Supportive care	*C. caretta*	14/39 (36%)	N/A	5–60	41–330	
[117] *	Supportive care	*L. kempii*	4/4 (100%)	N/A	N/A	30–45	
[154]	Supportive care	*L. kempii*	7/8 (88%)	>7	N/A	16–25	
[40]	Supportive care	*L. kempii*	5/6 (83%)	N/A	2–15	81	
[117] *	Supportive care	*C. mydas*	1/1 (100%)	N/A	N/A	30–45	
[154]	Supportive care	*C. mydas*	4/6 (67%)	>7	N/A	13–33	
[40]	Supportive care	*C. mydas*	2/7 (29%)	N/A	2–15	34–325	
[40,117,154]	Supportive care	*C. caretta*, *L. kempii*, *C. mydas*	39/75 (52%)	N/A	N/A	N/A	Combined data across sea turtle species.
[117] *	Supportive care + activated charcoal–kaolin suspension (5–10 mL/kg PO)	*C. caretta*	2/17 (12%)	N/A	N/A	49–61	
[138]	Cholestyramine (20 mg/kg or 50 mg/kg PO)	*T. scripta*	N/A	N/A	N/A	N/A	Failure to increase PbTx clearance; non-significant decrease in toxin concentrations in tissues.
[117]	Diphenhydramine (2 mg/kg IM q24h)	*C. caretta*	N/A	N/A	N/A	N/A	Successful treatment of conjunctival edema and prevention of corneal ulcers; little efficacy in reducing other symptoms.

^ This time may differ significantly, as several factors, such as the sustained presence of a harmful algal bloom in the area and the treatment of secondary conditions, may prolong this period despite successful brevetoxicosis treatment. ^#^ One turtle was treated with a dose of 100 mg/kg and did not show any side effects. * The loggerhead sea turtles in this HAB appeared to show symptoms that were not in line with prior experiences, potentially explaining disappointing treatment results. ^~^ Drastic change in neurological condition noted in 1–3 days, and sea turtles started eating on their own in 3–5 days.

## Data Availability

No new data were created or analyzed in this study. Data sharing is not applicable to this article.
